# Highly significant correlation between rates of adverse childhood experiences (ACE) in youth survivors of child sexual abuse (CSA) and their biological mothers

**DOI:** 10.1371/journal.pone.0340591

**Published:** 2026-01-12

**Authors:** Matthew Reeson, Hannah Pazderka, Wanda Polzin, Vincent Agyapong, Andrew J. Greenshaw, Yifeng Wei, Laurie Syzmanski, Peter H. Silverstone

**Affiliations:** 1 Department of Psychiatry, University of Alberta, 1E1 Walter Mackenzie Health Sciences Center (WMC), Edmonton, Canada; 2 Little Warriors Be Brave Ranch, Edmonton Alberta, Canada; 3 Department of Psychiatry, Dalhousie University, Abbie J Lane Building, Veterans Memorial Lane, Halifax, Nova Scotia, Canada; Tribhuvan University Institute of Medicine, NEPAL

## Abstract

**Background:**

Adverse Childhood Experiences (ACE) have been associated with poor long-term mental health outcomes. Measuring ACE scores in youth undergoing treatment, as well as their caregiver(s), may help inform treatment approaches and practice. This novel study examined a unique sample to attempt to help elucidate the relationship of trauma histories between youth survivors of child sexual abuse (CSA) and their biological mothers. Secondarily, we aimed to examine the distribution of ACE scores in order to evaluate if they demonstrate common underlying factors.

**Materials and methods:**

Children and adolescent CSA survivors aged 8–17 who were enrolled into a comprehensive multimodal 1-year treatment program for their abuse completed the Center for Youth Wellness ACE Questionnaire (CYW ACE-Q). Secondarily, a caregiver of each child was asked to complete the original 10-item ACE questionnaire (ACE-Q). Based on the available data, we report findings from a convenience sample of biological mothers of the CSA survivors. Biological mother ACE scores were statistically compared to determine if their trauma histories were associated with their child’s ACE score. Secondarily, a Principle Factor Analysis (PFA) was conducted to assess any underlying common factors among reported ACE scores.

**Results:**

A statistically significant moderate positive correlation was found between the biological mother and their child CSA survivor in terms of their ACE scores (r(107) = 0.56, *p* < 0.0001). Examining the underlying factor structure of the ACE scores, two factors (which we labelled *neglect* and *abuse*) accounted for the most variance in our sample (41.7%).

**Conclusions:**

The results of this study demonstrate a surprisingly strong, and highly statistically significant, correlation in the rates of high ACE scores between child CSA survivors and their biological mothers. These results strongly support the need for trauma-informed approaches when working with CSA survivors, and in particular all treatment approaches should also consider parental trauma histories. The findings of this study suggest that a family-centered, trauma-informed care approach should be considered, instead of only programming which is focused on the impacted child.

## Introduction

Brain development is a process that begins shortly after conception and continues into early adulthood [1]. Early-life toxic stress—when a child experiences strong, frequent, and/or prolonged adversity without adequate support—can impair brain development and increase the risk for stress-related disease and cognitive impairment [2,3]. Research on the biology of early-life stress has identified that chronic adversity during critical developmental periods can impair brain development and architecture [4,5]. Adverse Childhood Experiences (ACE) are defined as traumatizing events that occur before the age of 18. ACE are generally assessed using the ACE questionnaire (ACE-Q), which measures an individual’s exposure to toxic stress experiences from birth to 18 years of age. ACE have been consistently associated with a variety of negative psychiatric outcomes, including early onset mental illness and cognitive deficits [6–7]. The seminal ACE Study examined the connection between early-life adversity and long-term health outcomes in 18,000 adults, and showed that a higher level of exposure to early-life stress had a clear, dose-response relationship to an individual’s likelihood of developing physical, behavioral, and social problems in adulthood, as well as a higher likelihood of future victimization [8]. Subsequent studies have shown that higher ACE scores are associated with a variety of mental health issues, including depression, anxiety, substance abuse, suicidal ideation, and addiction [9–12], and can also impact parent-child relationships and healthy attachments [13–15].

Child Sexual Abuse (CSA) is a common ACE, and studies from the United States suggest approximately 8% of men and 16% of women have experienced at least one incident of sexual abuse prior to the age of 18 [16,17]. A recent meta-analysis on the prevalence of sexual violence among children reported a lifetime prevalence of 9.5% for girls and 5.5% for boys for any form of sexual contact or forced intercourse [18]. Rates of CSA in Canada are comparable, with an estimated 10% for males and 20% for females [19,20]. However, despite an increase in disclosure rates, a significant number of CSA incidents go unreported to the proper authorities; as such, these estimates undoubtedly understate the severity of the problem [21,22]. Of further consequence, there is evidence suggesting that there are particularly sensitive periods in childhood in which the deleterious effects of CSA can be amplified [23,24]. Some of this can potentially be mitigated, and research on resilience has shown that providing stable, responsive, nurturing relationships in the earliest years of life can prevent or even reverse the damaging effects of early life stress, with lifelong benefits for learning, behavior, and health [25–27].

Recent developments have begun to shed some light on the biological mechanisms involved in mediating traumatic experiences, some of which may involve epigenetic inheritance [2,28]. Contemporary research has suggested that the effects of childhood trauma may be transmitted to subsequent generations; in fact, a caregiver’s own unresolved trauma increases the risk that their child(ren) may be exposed to adverse events [29]. Mothers who were CSA survivors are more likely to have children who experience CSA; further, the presence of maternal CSA has been linked to psychological difficulties later in life for the child [30]. Children of CSA survivors are more likely to experience a higher level of childhood abuse and neglect when compared to those whose mothers did not have a CSA history [31]. Women sexually abused as children are at an increased risk for risky sexual behaviour and, consequently, teenage pregnancy, which can also lead to an increased risk of sexual victimization for children [32]. It has been well established that the effects of early-life stress on one individual can be passed down to subsequent generations [29,33,34]. In Canada, this is of particular significance to Indigenous populations. Bombay *et al.* [35] found greater depressive symptoms in the offspring of individuals who had been forced to attend Indian Residential Schools. Further research in these populations has shown an intergenerational link with alcoholism, suicide, and early death [36]. Using epigenetic markers, Yehuda *et al.* [37] found an increase in methylation in the functional region of the FKBP5 gene in the children of Holocaust survivors. The FKBP5 gene plays an important role in the regulation of the Hypothalamic-Pituitary-Adrenal (HPA) axis—a key pathway in stress regulation—and can be effectively disrupted through epigenetic modifications, such as methylation. Similarly, Yang and colleagues found altered methylation patterns at numerous sites for a group of maltreated children who were removed from their parents, compared to a group of age-matched controls [38]. Additionally, aside from genetic factors, caregivers may also transmit their unresolved trauma histories to their children via behavioural means, such as increased anxiety, unwillingness to allow developmentally appropriate behaviours such as exploration, and overreaction to hearing about their child’s negative experiences [28]. For these reasons, a better understanding of the transgenerational effects of ACE on mental health may help to develop preventative measures and alleviate these widespread problems. Furthermore, investigating the ways in which ACE pool into categories may elucidate a better understanding of the way certain ACE are related to specific behavioural and psychological outcomes.

Analyzing ACE data from both CSA survivors and their biological caregivers may provide a greater understanding of these relationships as well as determine best practices relating to intergenerational trauma. In this study, we present novel data collected from the Be Brave Ranch (BBR), a treatment facility specifically designed for youth CSA survivors [39–41]. As part of their regular intake activities, the BBR routinely collects self-report assessments, including ACE history. To our knowledge, no study has evaluated ACE histories in youth CSA survivors and their biological mothers. Using this novel data set, we aim to compare ACE scores from both youth and their caregivers. Before data collection, we hypothesized that there would be a statistically significant association between the ACE scores of CSA survivors and those of their biological caregivers. After data collection, the vast majority of respondents were biological mothers, so we limited the analysis to compare ACE scores in CSA survivors and their biological mothers. Secondarily, we conducted Principal Factor Analysis (PFA) to examine the relation of each ACE item among participants.

## Methods

### Study design

Here we present results from a convenience sample involving secondary use of de-identified data collected during routine admission to the Be Brave Ranch (BBR) program. Data for this study was collected on 219 youth CSA survivors and their biological caregivers from 01/01/2022–31/12/2024 based on data availability and the collection of data on an adequate sample size. As participants attend the program in a cohort sequential manner, gathering data on an adequate sample size required a longer study period. Of the 219 CSA survivors, 107 biological caregivers were able to complete the required surveys. Of the total sample collected, 44 (20.1%) participants had either CFS or foster care involvement and no biological caregiver was available to complete the surveys. Sixty-eight caregivers (31.1%) did not complete the survey. As a result, the 112 CSA survivors who did not have caregiver information were not included in the correlation analysis. From the 107 caregivers who did complete the survey, only 2 (0.9%) were completed by both the biological mother and father.

Participants were not directly recruited into this study; rather, the measurement-based care approach adopted by the BBR requires clients and their caregivers to complete standardized outcome measures to inform and evaluate programming. At intake into the program, all primary caregivers were required to complete written consent forms allowing for the collection of data for themselves and their child(ren). Consent forms were completed onsite with a staff member and included consent to conduct a secondary analysis of the data. Collected data was de-identified and provided to the research team for a secondary analysis of this collected data. No modifications were made in the data collection procedure during the study period. Ethics approval was obtained from the University of Alberta Human Ethics review board for this secondary use of the data (HREB ID Pro00089614).

### Participants

#### Youth CSA survivors.

Youth participants included in this study were children and adolescents admitted to a comprehensive 1-year multimodal treatment program specifically designed for child and adolescent survivors of CSA aged 8–17, the only one of its kind. Each participant underwent an initial screening interview conducted by a staff member to determine inclusion into the program. Individuals can be referred to the program through direct or online contact with program administrators. The criteria below are already put in place for program intake at the multimodal treatment program. These criteria were selected so that children can perform necessary self-care for daily living and can have the best chance of completing the full treatment program. Participants for the treatment program are required to:

(1) Have a history of child sexual abuse – disclosed to at least one adult
(2) Be aged 8–17
(3) Perform self-care necessary for daily living
(4) Have an IQ > 80 as determined by previous assessments. The program has certain psychoeducational components that require a functioning level above 80.
(5) Be mentally medically stable and compliant with prescribed medication. Medication are bubble-packed prior to admission into the program and are administered by trained staff on site.
(6) Have a family member/caregiver identified and involved
(7) Complete a readiness assessment which involves a screening interview by trained transition coordinators who determine eligibility and appropriateness for inclusion.

Exclusion criteria for children include:

(1) Severe mental health issues such as suicide attempt, self-harm, homicidal thoughts/actions, violent behaviors, and significant eating disorder. Actively in psychosis and/or not medically compliant with antipsychotic medication
(2) A significant runaway risk
(3) Serious behavioral problems
(4) Significant drug or alcohol use
(5) A history of sexual violence toward others
(6) Unstable medical conditions

#### Caregivers of CSA survivors.

Participants were recruited from a pool of caregivers who were positively involved in the treatment program and, as such, were not people who had been identified as perpetrators. Caregivers of CSA survivors in this study were fluent in English (based on observation when a staff team member assesses this at the time of intake), able to provide consent, and were caring for children who were enrolled in the multimodal treatment program. Non-biological parents (e.g., foster parents, CFS caseworkers, group home workers, etc.) were not included in this study as the intergenerational effects of ACE was the primary outcome under consideration. Further, biological fathers were not included as only two participants (0.9%) had both biological parents complete a survey and the sample size was not adequate to include these participants in the analysis. As such, to increase homogeneity of our sample, only biological mothers were included in this analysis. There were no explicit inclusion/exclusion criteria for the caregiver sample as mothers were not directly involved in treatment and were asked to complete surveys voluntarily.

### Outcome measures

Upon admission into the program, participants and caregivers are asked to complete a number of questionnaires which are used to guide individual treatment and to evaluate the efficacy of the program. Completion of these questionnaires is mandatory for program admission. To measure history of early-life trauma, primary caregivers were administered the ACE Questionnaire (ACE-Q), a 10-question self-report survey designed to measure an individual’s history of early-life adversity. Each question is a yes/no variable that asks about the individual’s history of a number of early traumatic life events. For the youth CSA survivors participating in the program, ACE history was measured using the Center for Youth Wellness ACE-Questionnaire (CYW ACE-Q). Both the ACE-Q and the CYW ACE-Q are considered a validated outcome measures for trauma histories [42]. The CYW ACE-Q is an 18-question survey that asks binary “yes/no” questions pertaining to a variety of common adverse events experienced by youth, including the standard 10 ACE items. Scores with an answer of “yes” are given 1 point and the total is summed. As such, higher scores indicate more ACE experienced by the individual. The CYW ACE-Q asks the participant about extra items outside the original 10 ACE, including experiencing bullying at school, living in foster care, being separated from a primary caregiver, and experiencing verbal or physical abuse from a romantic partner, among others. This questionnaire is designed specifically for youth and was given to the child and adolescent participants in this study. Given the association between ACE and the risk for mental health concerns, CYW ACE-Q score of >4 combined with symptomatology is generally considered a potential indicator for treatment referral [43].

### Statistical analysis

#### Descriptive statistics.

Demographic information was collected at the time of survey collection. Demographic information included participant age, sex assigned at birth, gender identity, Indigenous status, prior treatment for mental illness, prior pharmacotherapy for mental illness, and average annual household income.

#### Correlation analysis.

Correlation analysis was used to compare child and caregiver ACE scores. A correlation coefficient of r > 0.6 was considered strong, 0.4 < r < 0.6 moderate, and r < 0.4 weak [43]. Statistical significance was considered if p < 0.05.

#### Principal axis factor analysis.

The use of adverse events during childhood in research has shown to be highly dependable, as test-retest reliability in ACE questions has been assessed as good to excellent [44]. As such, we sought to explore if there was a more-or-less stable pattern of scores amongst survivors, potentially representing common factors underlying risk for sexual abuse at an early age.

To further examine the underlying structure of ACE scores in the patients, a Principal Axis Factor Analysis (PFA) with varimax rotation was performed (Table 3). Factor analysis is interested in investigating the underlying constructs of a given phenomenon [45]. In effect, PFA can determine which latent constructs drive responses by measuring interrelationships on our measured trauma variables. Varimax rotation was used in order to obtain orthogonal loadings for the factors. Analysis was limited to the 10 original ACE questionnaire items, less the sexual abuse question which was *de facto* experienced by everyone in the sample (i.e., all participants admitted for CSA treatment). The analysis passed the Kaiser-Meyer-Olkin Measure of Sampling Adequacy (.654), as well as Bartlett’s Test of Sphericity (<.001).

## Results

### Demographics

Demographic information for the CSA treatment participants and their caregivers are summarized in Table 1. In total, CYW-ACE data was collected for 219 youth CSA survivors (70 children and 149 adolescents). The average age of the child participants was 10.9 (1.5) years; for the adolescents 14.4 (1.3) years. Forty-six (65.7%) of the children and 143 (96.0%) of the adolescents were female assigned at birth (1 Because the adolescent program is limited to females for safety reasons, our data necessarily oversamples females); while 38 (54.3%) of the children and 121 (81.2%) of the adolescents identified with a female gender identity. Nine (12.9%) of the child participants and 23 (15.4%) of the adolescents identified with a non-binary gender. Twenty-three (32.9%) of the children and 63 (42.3%) of the adolescents identified as an Indigenous Canadian.

**Table 1:** Baseline participant demographics for primary caregivers and for youth child sexual abuse survivors receiving multimodal treatment for their trauma.

	Mothers (n = 107)	Children (n = 70)	Adolescents (n = 149)
Mean age (SD) (years)	38.4 (7.7)	10.9 (1.5)	14.4 (1.3)
Sex			
Female	107 (100%)	46 (65.7%)	143 (96.0%)
Male	0 (0%)	24 (34.3%)	6 (4.0%)
Gender Identity			
Female	107 (100%)	38 (54.3%)	121 (81.2%)
Male	0 (0%)	23 (32.9%)	5 (3.4%)
Non-binary	0 (0%)	9 (12.9%)	23 (15.4%)
Indigenous Canadian	35 (32.7%)	23 (32.9%)	63 (42.3%)
Previously treated for mental illness	67 (62.6%)	28 (40.0%)	79 (53.0%)
Previously medicated for mental illness	52 (48.6%)	7 (10.0%)	68 (45.6%)
Annual household income			
Under $30 K	45 (42.1%)		
$30–50 K	24 (22.4%)		
$50–100 K	26 (24.3%)		
$100–250 K	10 (9.3%)		
Over $250 K	2 (1.9%)		

From the total sample, one hundred and seven children had their corresponding biological mother completed an ACE-Q. As such, 107 child-mother pairs were available for correlation analysis. The average age of the biological mothers was 38.4 (7.7) years. Thirty-five (32.7%) identified as an Indigenous Canadian. Of the youth participants, 48.9% (107) had been previously treated for a mental illness, and 75 (34.3%) had been pharmacologically medicated for their mental illness. For the mothers, 67 (62.6%) had been previously treated for mental illness and 52 (48.6%) had been pharmacologically medicated. Nearly half (42.1%) of the caregivers reported an average family income of under $30 000/year, and only 12 (11.2%) reported an annual income over $100 000.

### Nature of the CSA

As part of the intake surveys, caregivers are asked a few questions about their child’s sexual abuse. The 107 biological mothers included in this survey reported that 88 (82.2%) had been sexually abused on multiple occasions. The reported mean age at first traumatic incident was 6.7 ± 3.3 years old. The vast majority of survivors (94.4%) knew their offender prior to the abuse. Seventy-five (70.1%) of abusers were adults, 27 (25.2%) were adolescents, and 5 (4.7%) of offenders were other children.

### Caregiver versus child ACE score

**Fig 1:**
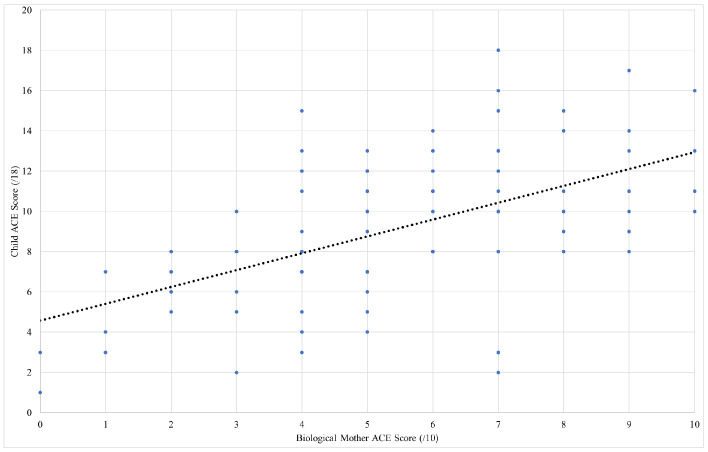
Linear correlation of CYW ACE-Q scores for youth child sexual abuse survivors compared to the ACE scores of their biological mothers (n = 107 matched pairs). The variables have a correlation coefficient of r(107) = 0.56 (*p* < 0.0001), which is considered a moderate correlation.

Correlation analysis was used to compare child and caregiver ACE scores. Fig 1 shows the correlation of child versus maternal ACE score. This analysis found a statistically significant correlation coefficient of r(107) = 0.56, (*p* < 0.001). Hence, this data supports the possibility that there is likely to be a strong to moderate correlation between ACE scores of children with CSA and their biological mothers.

### Distribution of ACE

The distribution of traumatic early-life events experienced by participants and caregivers is summarized in Table 2. As was expected, all youth participants reported having survived of sexual abuse; 54.2% of caregivers reported being sexually abused prior to the age of 18. Outside of sexual abuse, the most common ACE for the youth survivors were experiencing harassment or bullying at school (n = 182, 83.1%), experiencing verbal abuse (n = 171, 78.1%), living with a mentally ill household member (n = 170, 77.6%), and having parents who are separated or divorced (n = 169, 77.2%). For the mothers, the most commonly reported ACE were having parents who are separated or divorced (n = 76, 71.0%), surviving verbal abuse (n = 66, 61.7%), living with a household member with a substance use disorder (n = 60, 56.1%), and emotional neglect (n = 60, 56.1%).

**Table 2:** Prevalence of CYW ACE-Q items from a sample of child and adolescent CSA survivors and their biological mothers.

ACE Item	Youth CSA Survivors (n = 219)	Biological Mothers (n = 107)
Victim of sexual abuse	219 (100%)	58 (54.2%)
Victim of physical abuse	137 (62.6%)	55 (51.4%)
Victim of verbal abuse	171 (78.1%)	66 (61.7%)
Physically neglected	81 (37.0%)	32 (29.9%)
Emotional neglected	147 (67.1%)	60 (56.1%)
Parental separation/divorce	169 (77.2%)	76 (71.0%)
Witness intimate partner violence	126 (57.5%)	33 (30.8%)
Household mental illness	170 (77.6%)	56 (52.3%)
Household substance abuse	151 (68.9%)	60 (56.1%)
Incarcerated household member	89 (40.6%)	27 (25.2%)
Bullying and/or harassment	182 (83.1%)	
Death of a parent/guardian	42 (19.2%)	
Separation from primary caregiver	20 (9.1%)	
Serious/life threatening medical illness or procedure	104 (47.5%)	
Witness of violence in school or neighborhood	52 (23.7%)	
Incarcerated, detained, or arrested	75 (32.4%)	
Victim of racial discrimination	85 (38.8%)	
Victim of intimate partner abuse	83 (37.9%)	

**Note:** Percentages indicate the number of participants who answered “yes” to each of ACE on the CYW ACE-Q for youth child sexual abuse survivors undergoing multimodal treatment for their sexual abuse.

### Principal factor analysis

**Fig 2:**
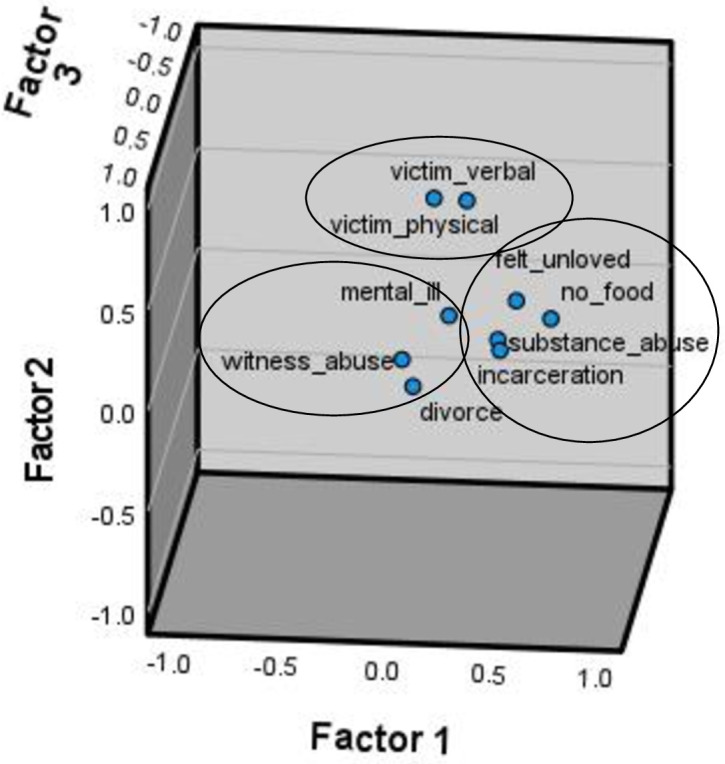
Factor Plot in Rotated Factor Space Illustrating Grouping of ACE Items among Child and Adolescent Survivors of CSA.

After the PFA was conducted, three factors were suggested given visual analysis of the scree plot as well as eigenvalues > 1.0. Table 3. displays the items and factor loadings for the rotated factors, with loadings less than.40 omitted for clarity. Upon examination, the three factors translated roughly as: *Neglect/abandonment*, characterized by not having food in the house, a parent who had been incarcerated and/or substance abusing, and verbal abuse; *Abuse*, characterized by either verbal or physical abuse; and *Family Breakdown*, which was marked by witnessing abuse, divorce, and (to a lesser extent) a cross-loading for incarceration. Note, however, that the accompanying interpretations for the factors are based upon subjective interpretation, and could reasonably be construed in other ways. Taken together, these three factors explain 53.76% of the variance in the data. These components are illustrated graphically by a biplot of the data, shown in Fig 2.

**Table 3:** Factor Loadings from Principal Axis Factor Analysis for the ACEs Questionnaire in Child/Adolescent CSA Survivors (*n* = 153).

Item	Factor Loading			Communality
	*Neglect*	*Abuse*	*Family Breakdown*	
More than once, you went without food, clothing, a place to live, or had no one to protect you	.671			.477
You often felt unsupported, unloved and/or unprotected	.488			.280
You lived with a household member who served time in jail or prison	.461		.348	.341
You lived with someone who had a problem with drinking or using drugs	.437			.241
A household member swore at, insulted, humiliated, or put you down in a way that scared you OR a household member acted in a way that made you afraid that you might be physically hurt		.752		.603
Someone pushed, grabbed, slapped or threw something at you OR you were hit so hard that you were injured or had marks		.614		.485
You saw or heard household members hurt or threaten to hurt each other			.451	.207
Your parents or guardians were separated or divorced			.375	.154
You lived with a household member who was depressed, mentally ill or attempted suicide				.152

**Note:** Loadings < .40 are omitted.

We also sought to assess the internal consistency of each of these factors, to determine whether they are measuring the same underlying construct. While Cronbach’s alpha values over.70 are generally considered desirable, it has been observed that when there are a small number (fewer than 10) of items in each scale, scores on this measure may be lower than expected. As such, scores above.50 are generally considered acceptable, but should be reported alongside mean inter-item correlations.

The first two factors each displayed adequate internal consistency. Cronbach’s alpha for *Neglect* was.612, with a mean inter-item correlation of.281. Similarly, Cronbach’s alpha for *Abuse* was.633, with a mean inter-item correlation of.465. Both of these factors could be described as fairly robust, considered the low number of variables in each factor (four and two, respectively). However, the same could not be said of the third factor, *Family Breakdown*, which had a Cronbach’s alpha score of only.349, and mean inter-item correlation of.153. As a result, we suggest only the first two factors should be regarded as underlying constructs of trauma amongst survivors of CSA.

## Discussion

### Distribution of ACE scores and implications

The results from the present analysis support our hypothesis that there would be a statistically significant positive correlation between youth child sexual abuse (CSA) survivor ACE scores and those of their biological mothers. These findings support the current evidence that the risk for CSA is higher in children whose parents have a significant history of early-life trauma [30,31]. Although an association between early-life trauma in biological caregivers and children been established in other literature [2,33,46,47], this study provides the first collection of trauma histories from sample of youth CSA survivors and their biological mothers. From this data, we found that the number of ACE reported by both caregivers and their children were significantly higher than the surveyed general population [48,49]. The high prevalence of these ACE in this population supports the notion that CSA survivors are 2–3 times more likely to experience other forms of trauma as compared to those with no history of CSA [50]. Unfortunately, to our knowledge, no Canadian data regarding the prevalence of ACE in youth, as measured by the CYW ACE-Q, currently exists. The 2014 US National Survey of Children’s Exposure to Violence collected ACE scores for 1,949 children and adolescents aged 10–17 [48]. In this study, the reported prevalence of each ACE item was significantly lower for than in our sample. Notably, our sample reported significantly higher rates of physical abuse (62.6% vs. 13.3%), verbal abuse (78.1% vs. 13.5%), and emotional neglect (67.1% vs. 6.4%). Of the nearly 2,000 youth sampled in the Us National Survey, 11.8% of respondents reported 5 or more of the original 10 ACE items; in our sample, 78.6% reported experience 5 or more ACE. This highlights the way in which ACE often pool in certain populations. An unexpected finding from our analysis was the high proportion of mothers who are survivors of CSA themselves, with over half reporting some form of sexual abuse prior to the age of 18. A comparison sample of over 44,000 adult ACE scores from the Canadian Longitudinal Study on Aging reported a prevalence of 17.3% for CSA compared to 54.2% of our sample [49]. The frequency of ACE reported in the biological mother sample was notably higher for all the ACE items collected, primarily parental separation and divorce, victim of physical abuse, and witness of domestic violence, as compared to other adult ACE studies [50]. A recent meta-analysis of ACE prevalence worldwide found that roughly 40% of the population has experienced no ACE and 16% have experienced more than 4 [51]. A similar recent study of over 200,000 American adults found a mean ACE score of 1.56, further highlighting the observed disparity [52]. In our sample, only two mothers reported an ACE of 0 (1.9%) and the median ACE score was 5. For the child and adolescent participants, the median score on the first 10 items of the CYW ACE-Q was 7. In other words, both the caregivers and the youth survivors showed scores several times higher than multiple comparison populations, and appears to be increasing intergenerationally.

### Findings of the principal axis factor analysis

The principal axis exploratory factor analysis conducted in this study revealed three factors associated with a history of CSA: *neglect, abuse*, and *family breakdown*. Neglect—which included reports of having endured long periods without food or shelter, having a family member who was incarcerated, having a family member with a substance use disorder, and feeling unloved—accounted for the highest explained variance (21%) in the youth surveyed, which may suggest that childhood neglect is a greater risk factor for CSA than other ACE factors. Children who are left to care for themselves are more susceptible to sexual abuse as their caregivers are often absent and unable to protect them from potential offenders [53]. They may also feel less comfortable turning to available adults with their concerns. Importantly, these findings also call into question the tendency to lump abuse and neglect together, which remains common practice despite evidence suggesting the experiences are very different and result in distinct consequences. As Golden et al. [54] notes, “Most think about abuse and neglect as if they go together. They are linked in our language and legislation as well as in our minds.” Neglect involves a situation in which there is a failure to meet the emotional, physical, and health needs of the child, rather than bullying, intimidation, or overt acts of violence, making it materially different from abuse. Although there is some evidence for an increased risk of CSA in children of parents with substance use disorder (SUD) or severe mental illness, the relationship is complicated by the identity of the offender—be it the affected parent or someone else [55]. However, children whose parents have a lifetime diagnosis of a SUD are more likely to suffer more severe neglect [56], which may also increase their vulnerability to sexual abuse due to parent absenteeism. Furthermore, children of substance abusive parents with high ACE scores are more likely themselves to suffer from a number of ACE, including physical and verbal abuse, highlighting the transgenerational and cyclical transmission of early-life stress and mental illness [12].

The factor analysis also revealed an association between CSA and either verbal/physical abuse, (*abuse* factor), and with divorce, incarceration, and the witnessing of abuse (*family breakdown* factor)—although these were relatively weaker than the *neglect* factor, each accounting for 15.6% and 12.1% of the variance, respectively. However, upon closer examination, the family breakdown factor showed poor internal consistency. This is likely because the items comprising the factor (divorce, witnessing abuse, and incarceration) may or may not co-occur. The finding that neglect explains more of the variance than abuse is perhaps counterintuitive, as it appears common to think of neglect as being somehow benign, but our analysis suggests that, when it comes to CSA, this is not the case.

Differentiating early-life traumatic experiences by subtype may be useful in assisting health professionals to determine which psychopathologies an individual is most susceptible to, based on their trauma history [57]. Recognizing that both neglect and abuse play a role in CSA, it may be useful to consider the differentiating impacts of neglect and abuse on long-term outcomes and the way in which a specific ACE may manifest psychologically. For example, emotional triggers may differ for an individual who was physically neglected (e.g., hunger) as opposed to an individual with a history of verbal abuse (e.g., loud noises). Understanding patterns of ACE scores likely to be seen in CSA survivors may offer fruitful suggestions for treatment approaches.

### Implications for policy and program development

These results highlight the way in which ACE can disproportionately impact and accumulate in certain populations, potentially making subsequent generations more vulnerable to other forms of early-life trauma. These findings also emphasize the importance of trauma-informed education for both CSA survivors and their caregivers. Trauma-informed developmental care within children’s mental health and treatment programming has been defined as best practice [58]. This includes, but is not limited to, consideration of the child’s *developmental* age versus *chronological* age, identifying and addressing trauma in its earliest stages wherever possible, and addressing care plans and service delivery based upon mindful reflection of how trauma impacts people’s lives, their family system, as well as their unique service needs [59]. Trauma-informed approaches emphasize the three pillars of trauma-informed care, which are: (1) establishing a sense of safety; (2) enhancing coping skills to manage emotions; and (3) promoting positive social connections [60]. Trauma-focused family systems intervention and care could be a useful addition to treatment program design. Support for caregivers is important to help them learn to be self-reflective, attachment based, and trauma-informed in their parenting styles, so that intergenerational trauma cycles are not unintentionally passed from caregiver(s) to their child(ren) [61]. Further, mental health supports should be considered within the context of the family system whenever early childhood trauma and early childhood sexual abuse is identified [62]. This is in contrast to current practice which treats child and parent (or caregiver) separately, usually without support for the family as a whole [63].

These findings emphasize that it may be more appropriate to incorporate dyadic and/or family systems therapy into standard treatment for children with developmental trauma disorder, with a particular eye to potential shared trauma histories. It has become evident that family-centered, trauma-informed approaches with the child as well as their caregiver(s) is necessary and invaluable [64]. As there is a complex interplay between the trauma narratives of both the child and caregiver, how those narratives affect their present can impact their response to therapy. For example, the trauma history of the parent or caregiver may interfere with a child’s treatment, via emotional and behavioral strategies that reflect issues with the adults’ coping with their own trauma rather than the current functioning of the child. This family-centered approach necessitates involving a key caregiver in the treatment, recognizing the crucial role of the caregiver in helping to provide a safe environment for the child, and giving them the tools to resolve their own issues. This approach may also delve into the caregiver’s history and *their* need for clinical interventions and supports, something which our current healthcare structure does not advocate. In so doing, we recognize the patient as the *family unit* versus any one individual. Family programming and caregiver supports function under the belief that a family-centered, trauma-informed approach can be integrated into clinical practice and policy by expanding service beyond the individual child/ teen programming to include targeted family psycho-education and therapy. Teaching caregivers how trauma impacts the entire family unit and identifying and interrupting dysfunctional patterns can reduce stress, improve communication and foster resilience. Additionally, including the family and viewing them as integral to healing helps normalize for the client that they are not alone and that healing doesn’t always happen in isolation. Evidenced-based strategies such as Emotion-Focused Family Therapy and tailored family therapy sessions help caregivers respond more effectively to their child’s emotional, behavioural and mental health needs, while also addressing their own caregiver blocks [64,65]. This model strengthens family functioning and enhances treatment outcomes for the individual client. For optimal outcomes, treatment best practice should be delivered by an integrated team who have a broad understanding of how to help both the child survivor and their caregiver and/or family.

In this context, the ACE-Q and the CYW ACE-Q are valuable tools for assisting the clinical team in identifying the past narrative context, and how those narratives may influence the present story for both the caregiver and child. Trauma-informed family care and intervention supports the belief that traumatic experiences are relatively common and that people and families often encounter numerous adverse experiences throughout their lifetime. This assertion is further supported by the growing literature noting the association of parental ACE as a risk factor for trans-generational childhood adversity [33]. Epigenetic research has elucidated the potential biological mechanisms that mediated this intergenerational transmission of trauma [66]. There is evidence to suggest that survivors of traumatic experiences can pass down heritable predispositions to stress dysregulation and psychopathology [37,67]. In fact, the finding that parental neglect is the factor explaining the greatest amount of variance in our sample may reflect that parents with high levels of ACE themselves may be challenged in providing adequate care to their offspring. Accordingly, maternal trauma history accounts for up to one-third of the variance in predicting child maltreatment, and may therefore be a better predictor of child ACE score [68]. In working with mental health and addictions therapists, children and families can present with a complex range of symptoms which have not previously been connected with their trauma history [69]. By identifying the ACE score of both child and caregiver, trauma-informed intervention and treatment can engage with a family-focused approach. Understanding ACE history may enhance caregiver empathy towards their child as they reflect on their own experiences and develop a better connection to their child [70]. Effective programs to support caregiver relationships are key to the successful emotional, social, and cognitive development of children [64]. The results from the present analysis demonstrate that the ACE score of adult caregivers may provide helpful information for families and clinicians.

### Limitations

There were a few limitations that need to be kept in mind in interpreting our findings. Firstly, there was no direct control group to compare to those in the program or their caregivers. Specifically, it would have been helpful to have data from a non-CSA population, gathered at the same time and using the same methods, to compare directly against. Secondly, ACE scores for caregivers encompass the first 18-years of their lives; as the children in the program had an average age of 12, their scores may not adequately represent a complete ACE history—which makes their elevated scores that much more significant. Although both these measures are intended to measure trauma history, the complex nature of trauma is inevitably difficult to fully capture through surveys or scales, which should be considered when interpreting these findings. It should be noted that, since all of the children admitted to the program are survivors of sexual abuse, and because sexual abuse is one of the ACE included in the ACE-Q, *a priori* none of the youth participants could have a CYW ACE-Q score of zero. Finally, our convenience sample was limited to biological mothers as there were too few participants representing other types of caregivers (biological fathers, foster parents, care providers, etc.). This limits the generalizability of the findings to any caregiver of CSA survivors; however, we felt including data from non-biological mothers would have introduced significant heterogeneity into the sample. Gathering data on biological fathers as well as mothers may elucidate further findings in regard to dyadic trauma histories. Larger sample sizes that include a variety of caregivers would provide better clarity on the link between caregiver and child ACE scores in the context of CSA.

### Future directions

The findings of this study demonstrate the need for future research to look beyond treating child developmental trauma in isolation. Contemporary research on trauma-informed care and leading clinical practices encourage an approach to treat the family system as a whole [71]. There continues to be a significant need to look at risk factors for ACE in an attempt to mitigate their prevalence. Future research should also try and identify resiliency factors alongside the collection of ACE scores. At the same time, improving policy-makers’ understanding of the significance of ACE is imperative to improving awareness and driving effective research. Finally, when working in the area of developmental and generational trauma, encouraging therapists to work from a trauma-informed, family-centered lens is essential.

Additionally, future research should directly examine whether the unresolved parental/caregiver trauma can interfere with the treatment goals of the child, thereby altering the trajectory of healing. Evidence informed outcomes should be measured for child, dyad, and family. Furthermore, funding allocations based on a positive outcomes approach, instead of the current situation where funding is allocated solely based upon the number of individuals treated, would be optimal. By collecting both child and caregiver ACE, early targeted interventions for children at a high risk for toxic stress, adversity, and disrupted attachments, can be developed.

## Conclusion

The key finding from the present analysis is that biological caregivers for survivors of CSA may themselves have experienced multiple types of trauma in their own childhoods, including CSA. From this standpoint, it is important to recognize that ACE, as they relate to an individual child, cannot be understood in isolation. The interplay between the child, their caregiver(s), and the combined history of adverse events, invokes a necessary conversation for healing and treating developmental trauma. In clinical practice, some protective factors for supporting children and adolescents with their developmental trauma comes within the context of treating the entire family; understanding this highlights the importance of factors that predict resilience.

In many jurisdictions the mental healthcare system is separate for children and for adults, with little to no integration of the parent-child dyad. Specialized services tend to focus on a specific population due to funding limitations and implications. Yet high correlations in intrafamilial ACE demonstrate the transmission of trauma and adversity. These results support clinical observations suggesting that there may be an intergenerational relationship between trauma experienced by the parent (and/or caregiver) and that of the child. Our conclusion from this analysis is that the status quo in treating childhood developmental trauma needs to shift towards a seamless family system approach. It is believed that this integrated approach would better address the longer-term term negative mental health and addictions symptoms that are tightly associated with childhood abuse.
